# Account of an Abscess of the Liver, Terminating Favorably by Evacuation through the Lungs

**Published:** 1807-06

**Authors:** F. Pascalis

**Affiliations:** Philadelphia


### *Account of an* Abscess *of the* Liver, *terminating favorably by Evacuation through the Lungs.* By F. Pascalis, m. d. *of Philadelphia.*

THE history of successful treatment in a dangerous complaint should not be indifferent to medical readers; but the following case will, perhaps, appear the more interesting, as we may derive from it practical means for discriminating between distinct causes and similar effects, and for a method of cure far different from ancient and much approved doctrines.

The patient I allude to, a man of forty-two years of age, is evidently of a sound constitution and of a regular mode of living; yet, with a bilious habit, and a great share of organical irritability, he had much impaired his health by previous fatigue of mind and body, when, in the middle of last July, he was seized with a violent fever and delirium. No time was lost to ascertain the nature of his attack. Three eminent physicians endeavoured to counteract the inflammatory symptoms by repeated bleedings and laxative remedies. Two days elapsed without any abatement of the fever; and it seemed still difficult to give a name to the disease, as no particular determination could yet be pointed out. Five times the blood had offered offered the appearance of a *lotura carnium.* On the third night, however, the patient began to cough, and to spit much mucus tinged with blood. He complained also of a pain beneath the right breast, which extended down in the hypochondre of the same side, and connected with the shoulder. A tumour was now discovered on the liver, and felt below the true ribs; for this reason, depleting and antiphlogistic remedies were continued, with blisters and cuppings on the affected parts, and two grains of calomel every hour or two; but the stomach was so irritable that it frequently rejected this and other medicines, which had very little effect or none at all;—it was, therefore, by the help of six successive bleedings more, that the patient apparently grew better, and could at the end of three weeks sit up and walk a little. We must now observe, that during the abatement of the inflammatory symptoms, respiration was sometimes tremulous, a slight yellow suffusion had spread over the body; night sweats were profuse, and the patient could not lie down any length of time without being roused up by horrid feelings of an impending suffocation, and great was besides the bodily anxiety, which proved his convalescence to be delusive; indeed, a fresh pulmonary inflammation took place, with cough, violent delirium, and fever. The exacerbations were great, in the evening especially, and required three successive bleedings within the period of four days. On the fifth, the case was really much to be despaired of; the debility being extreme, the cough unabated, the pulse corded, and the night sweats rendered very distressing by the heat of the season. The night following he was seized with such spasms of the bronchiæ, that he would have attempted to bleed himself, had he not been promptly relieved by that operation. The emergency of the case pointed out to the attending physician, that as an internal abscess could not be doubted of, he must stimulate and even convulse the primæ viæ, to promote its rupture and discharge. A light antimonial mixture evidently contributed to that desirable effect; for after several doses were taken, large evacuations of pus were brought on, by and with a cough. They continued as it were by spells, and took place, once at least, in twelve hours. They were also easily promoted by taking nourishment, or tonics, and by the jolting of a carriage; the nauseous taste of the matter frequently excited vomiting, which altogether, with the cough, rendered the convulsive exertions of the abdominal muscles very distressing. These evacuations lasted nearly eight days, and and after the two last, which were more copious and mixed with white flakes, the patient, who was already altered for the better, remained convalescent. A slight soreness was felt in the right hyponchondre, which gradually disappeared. By the help of a sound and restrained diet, and of exercise by riding, he has attained a perfect state of health.

We may distinctly perceive three periods in this case; that of an inflammatory fever preceding the formation of an abscess in the liver, and attended with a symptomatic pulmonary inflammation; that of a cessation of all inflammatory symptoms, offering evidently those of an internal imposthume; and that of a fresh inflammation in the diaphragm and lungs, immediately followed by the discharge of the matter. It therefore could not be a subject of doubt whether the abscess was in the liver, since the patient began to feel pain, to cough, and to spit blood, on the third day only of the disease. The latter symptoms, indeed, should have kept pace with the invasion of the fever, had the pulmonary inflammation been the primary disease. The tumour or hardness discovered and felt under the true ribs, and the yellowness of the skin, were also direct proofs of the affection of the liver, the subsequent effects of which were evinced by the existence of the cough only, during the first inflammation of that viscus, and at the period of the discharge of the suppurated fluids through the diaphragm and lungs. The possible inflammatory adhesion of those parts is a well-established point of pathological doctrine, and of course they may simultaneously be corroded by the pus, and open an easy issue through the air vesicles and bronchiæ. Lieutaud mentions the instance of a monstrous tumour on the abdomen, which proved to be an abscess of the liver, when it was discharged through the lungs, and almost emptied before the exhausted patient expired. Indeed, a fortunate concourse of many circumstances only can determine such a favourable termination of the Pleuritica-hepatitis, as Cullen observes; but it may be encouraging to remark, that instances of the same are not uncommon.

No case of disease, I should believe, requires a more sagacious and experienced judgment in practice. Contrary to what we have been taught by many celebrated writers, to withhold the lancet after suppurative inflammation, in the present instance we see, that it has been *fifteen times* resorted to, and with success. I cannot say the same of mercury, although it is almost a specific in hepatitis; hepatitis; for our patient, who was repeatedly put under its operation, received no benefit from it, perhaps owing to the rapid formation of the abscess, or to accidental insufficiency of that medicine. Tonic and stimulating remedies were of great help, and in no case of continual exhaustion have I witnessed more advantage derived from nourishment, sparingly, but frequently exhibited.

Before I conclude this paper, permit me to inform the Reader that I was myself the patient alluded to, and to offer my sincere and unfeigned thanks to the three eminent physicians who conducted my treatment, Dr. Rush, Dr. Physick, and Dr. Caldwell. Their sedulous kindness, guided by a skilful judgment, adopted a mode of practice apparently violent; but it was evinced the wisest, in a case which offered no chance from nature or constitution. Of the attending physician, Dr. Rush, who had the most troublesome task, during seven weeks of confinement, I should not forget to mention the humane and affectionate care; nor to remark, that with his great talents, he eminently possesses all the qualities which constitute the physician. I wish for those gentlemen, all possible professional success and private happiness.

*Sept.* 28, 1804.

